# Patterns of distant metastases in patients with clear cell renal cell carcinoma––A population‐based analysis

**DOI:** 10.1002/cam4.3596

**Published:** 2020-11-28

**Authors:** Jianxin Xue, Wensun Chen, Wenbo Xu, Zicheng Xu, Xiao Li, Feng Qi, Zengjun Wang

**Affiliations:** ^1^ Department of Urology The First Affiliated Hospital of Nanjing Medical University Nanjing China; ^2^ Department of Urology Nanjing Hospital Affiliated to Nanjing University of Chinese Medicine the Second Hospital of Nanjing Nanjing China; ^3^ First Clinical Medical College of Nanjing Medical University Nanjing China; ^4^ Department of Surgery The Fifth Affiliated Hospital of Zhengzhou University Zhengzhou Henan China; ^5^ Department of Urologic Surgery Jiangsu Cancer Hospital & Jiangsu Institute of Cancer Research & Affiliated Cancer Hospital of Nanjing Medical University Nanjing China

**Keywords:** metastasis, prognosis, renal cancer, SEER

## Abstract

We developed this study to describe the patterns of distant metastasis (DM) and explore the predictive and prognostic factors of DM in clear cell renal cell carcinoma (ccRCC) patients. We collected the eligible patients from the Surveillance, Epidemiology, and End Result (SEER) database from 2010 to 2015. Then, comparisons of baseline characteristics between patients in different metastatic patterns were made. In addition, proportional mortality ratios (PMRs) and proportion trends of different patterns were calculated. Afterward, survival outcomes were explored by Kaplan–Meier (KM) analyses. Finally, predictive and prognostic factors of DM were investigated. A total of 33,449 ccRCC patients were eventually identified, including 2931 patients with DM and 30,518 patients without DM. 8.76% of patients suffered DM at their initial diagnosis, 35.01% of them had multiple metastases. Generally, lung (6.19%) was the most common metastatic site in patients with DM, and brain (1.20%) was the least frequent metastatic organ. The proportion trends of different metastatic patterns tended to be stable between 2010 and 2015. Moreover, higher tumor grade, T stage, and N stage were identified as risk factors of DM. Finally, age at diagnosis, grade, T stage, N stage, the administration of surgery, the number of metastatic sties, marital status, and household income were found to be significantly associated with prognosis. Lung was the most common metastatic site in ccRCC patients. Different survival outcomes and prognostic factors were identified for different metastatic patterns. Hence, our study would have great value for clinical practice in the future.

## Graphical abstract

Lung was the most common metastatic site in ccRCC patients. Different survivals and prognostic factors were identified for different metastatic patterns. Hence, our study would have great value for clinical practice in the future.

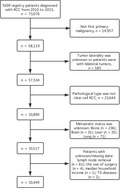

## 1 INTRODUCTION

Kidney cancer is one of the most common malignancies of urinary system, second only to bladder cancer. The latest research revealed that the estimated new cases and deaths are 73,750 and 14,830 in 2020 in the United States.^1^ Renal cell carcinoma (RCC) accounts for about 90% of all kidney malignancies, and is mainly composed of clear cell RCC (ccRCC), papillary RCC, chromophobe RCC, and so on.^2^, ^3^ Among them, ccRCC is the most common subtype, responsible for 70% of all RCC cases.^4^, ^5^

Previous studies have reported that up to 18%–30% of RCC patients were with systemic metastases at the time of initial diagnosis, and another third progressed to metastatic diseases after nephrectomy during the long‐term follow‐up.^6^, ^7^ Generally, patients with advanced or metastatic RCC (mRCC) have poor prognosis, with a median overall survival (OS) of about 13 months.^8^ Recently, the 5‐year OS increased slightly from 7.3% to 12.3%.^9^ Despite the distant metastasis (DM), many studies have confirmed that mRCC patients could benefit from cytoreductive nephrectomy (CN),^10^, ^11^, ^12^ and CN has been the standard treatment since 2001.^13^, ^14^ Guo et al.^15^ explored the value of CN among RCC patients with liver metastasis, and they demonstrated that CN prolonged the OS in this population. Lin et al.^16^ investigated the role of surgical intervention on RCC patients with lung and bronchus metastasis, they found that these patients could obtain better prognosis after having surgical intervention than those without surgery.

Lung was considered to be the most common metastatic site in patients with ccRCC, followed by bone.^16^, ^17^ Previous studies concluded that the 5‐year OS rates for patients with lung metastases received pulmonary metastasectomy varied from 36% to 83%. Ljungberg et al.^18^ demonstrated that antiangiogenic therapy was strongly recommended in mRCC patients without choice of further surgical treatment, while its actual efficacy was limited, with a median OS of 26.4–32.0 months for those patients.

Considering the high rate of metastatic diseases and the poor prognosis of mRCC patients, it was of great value to investigate the predictive and prognostic factors of DM in ccRCC patients. However, most of previous studies were single‐center, with small sample size, and without long‐term follow‐up. Hence, we developed this study on the basis of the Surveillance, Epidemiology, and End Results (SEER) database to investigate the risk factors of DM and the prognosis of mRCC patients with different metastatic patterns.

## 2 METHODS

### 2.1 Database

All data used in this study were downloaded from the SEER database retrospectively. SEER registry is a public database collects the detailed information of all cancer patients, including incidence rates, basic characteristics, treatment, mortality, and long‐term follow‐up outcomes. Initially, there were only nine regions in this project. However, with increasing regions take part in this program, the SEER 18 covers approximately 30% of the whole U.S. population. For this study, we signed the data agreement and utilized the SEER database with the username 15440‐Nov2018. Moreover, the application of SEER database was exempt by Institutional Review Board approval.

### 2.2 Patient identification

In this study, patients diagnosed with ccRCC from 2010 to 2015 were retrospectively extracted from SEER 18 using the SEER* Stat software (Version 8.3.6; NCI). The inclusion criteria were as follows: (a) patients diagnosed with ccRCC with positive pathology (C74.9, International Classification of Diseases for Oncology: 8310/3), (b) patients with active follow‐up and complete data, (c) ccRCC was the first primary malignancy. Furthermore, patients met any of following criteria should be excluded: (a) tumor laterality was unknown or patients with bilateral tumors; (b) metastatic status was unknown (including brain, bone, liver, and bone); (c) missing/unknown data on the administration of surgery, lymph node removal, median household income, and so on; (d) reporting source was autopsy/death certificate only.

### 2.3 Data extraction

Baseline characteristics and follow‐up outcomes were collected for each eligible patient utilizing the “Case Listing Session” in the SEER*Stat software, variables including age at diagnosis, gender, race, year of diagnosis, tumor laterality, grade, American Joint Committee on Cancer (AJCC) 7th T stage, N stage, the administration of surgery and lymph node removal, metastatic status, vital status, survival months, cause of death (COD), insurance status, marital status at diagnosis, and median household income. All the enrolled patients were divided into two groups (With DM and Without DM) depending on whether the patients had DM. Additionally, patients with DM were further categorized based on the number and patterns of metastatic sites.

In our study, age at diagnosis was classified into <65 and ≥65 years old. Race was divided into White, Black, and Other (including American Indian/AK Native, Asian/Pacific Islander). Tumor grade was categorized into Grade I (well differentiated), Grade II (moderately differentiated), Grade III (poorly differentiated), and Grade IV (undifferentiated). High and low levels of household income were defined according to the median value. Moreover, in order to investigate the death patterns of died patients, we divided the COD into “RCC,” “cardiovascular disease (CVD),” and “other cause.”

### 2.4 Incidence of distant metastasis, proportional mortality ratio

To study the trends of DM in recent years, we calculated the specific proportions of different groups in newly diagnosed cases. Besides, further subgroup analyses were performed on the basis of the number and patterns of metastatic sites.

Proportional mortality ratio (PMR) was calculated as the death number due to a specific cause divided by the number of deaths in the whole population. Similarly, we compared the PMRs of RCC, CVD, and other cause between ccRCC patients with or without DM, and further stratified by the number and patterns of metastatic sites.

### 2.5 Survival outcomes

Kaplan–Meier (KM) analyses were constructed to investigate the long‐term survival outcomes of ccRCC patients. Then, univariate and multivariate logistic/Cox regression analyses were developed to explore the predictive and prognostic factors of DM.

### 2.6 Statistical analysis

Chi‐square test was used to make comparisons of categorical variables between different groups. Cancer‐specific survival (CSS) or OS curves were presented by utilizing KM plots. The complete analyses were performed via SPSS 23.0 software (SPSS Inc) and R software (Version 3.4.1). All analyses were two‐sided and *p *< 0.05 was considered to be statistically significant.

## 3 RESULTS

### 3.1 Baseline characteristics and survival outcomes

**FIGURE 1:**
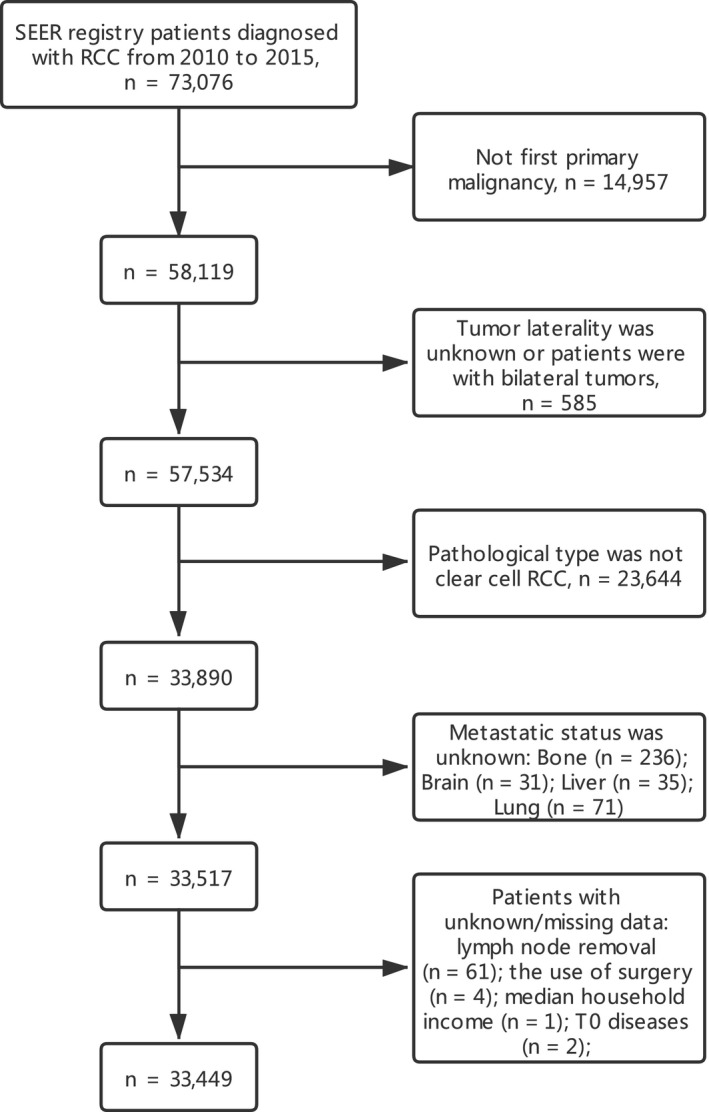
Flowchart of patient selection

The flowchart of patient selection is shown in Figure 1. As shown in Table 1, a total of 33,449 ccRCC patients were eventually identified in this study, including 2931 patients with DM and 30,518 patients without DM, with an average age of 60.10 years old. In general, most patients were male (62.38%), White (84.59%), with early stage of diseases (T1: 65.02%, N0: 95.06%, localized histology: 72.13%), and cancer‐directed surgery (94.05%). When compared with patients without DM, those with DM had an older age (mean: 62.29 vs. 59.89 years, *p* < 0.001), higher probability of male (70.62% vs. 61.68%, *p* < 0.001), and later stage of diseases (T3‐4: 56.23% vs. 19.84%, *p* < 0.001. N1: 24.26% vs. 1.55%, *p* < 0.001). Furthermore, the rate of surgery was significantly higher (97.63% vs. 56.67%, *p* < 0.001) while lymph node removal rate was significantly lower (10.47% vs. 21.22%, *p* < 0.001) in patients without DM. However, no significant difference was detected in the comparisons in tumor laterality (*p* = 0.127) and median household income (*p* = 0.597).

**TABLE 1:** Baseline characteristics of included patients

	Total	Without DM	With DM	*p* value
N	33,449	30,518	2931	
Age, y, Mean±SD	60.10±12.30	59.89±12.42	62.29±10.78	<0.001
<65	20,723 (61.95%)	18,999 (62.26%)	1724 (58.82%)	<0.001
≥65	12,726 (38.05%)	11,519 (37.74%)	1207 (41.18%)	
Sex				
Male	20,864 (62.38%)	18,794 (61.58%)	2070 (70.62%)	<0.001
Female	12,585 (37.62%)	11,724 (38.42%)	861 (29.38%)	
Race				0.001
White	28,293 (84.59%)	25,794 (84.52%)	2499 (85.26%)	
Black	2421 (7.24%)	2235 (7.32%)	186 (6.35%)	
Other	2453 (7.33%)	2217 (7.26%)	236 (8.05%)	
Unknown	282 (0.84%)	272 (0.89%)	10 (0.34%)	
Laterality				0.127
Left	16,406 (49.05%)	14,929 (48.92%)	1477 (50.39%)	
Right	17,043 (50.95%)	15,589 (51.08%)	1454 (49.61%)	
Grade^a^				<0.001
Grade I	3297 (9.86%)	3235 (10.60%)	62 (2.12%)	
Grade II	15,438 (46.15%)	14,989 (49.12%)	449 (15.32%)	
Grade III	8314 (24.86%)	7497 (24.57%)	817 (27.87%)	
Grade IV	2001 (5.98%)	1457 (4.77%)	544 (18.56%)	
Unknown	4399 (13.15%)	3340 (10.94%)	1059 (36.13%)	
Histology				<0.001
Localized	24,128 (72.13%)	24,128 (79.06%)	0 (0.00%)	
Regional	5882 (17.58%)	5882 (19.27%)	0 (0.00%)	
Distant	3339 (9.98%)	408 (1.34%)	2931 (100.00%)	
Unstaged	100 (0.30%)	100 (0.33%)	0 (0.00%)	
T stage				<0.001
T1	21,749 (65.02%)	21,278 (69.72%)	471 (16.07%)	
T2	3493 (10.44%)	2950 (9.67%)	543 (18.53%)	
T3	7128 (21.31%)	5827 (19.09%)	1301 (44.39%)	
T4	576 (1.72%)	229 (0.75%)	347 (11.84%)	
Tx	503 (1.50%)	234 (0.77%)	269 (9.18%)	
N stage				<0.001
N0	31,797 (95.06%)	29,802 (97.65%)	1995 (68.07%)	
N1	1184 (3.54%)	473 (1.55%)	711 (24.26%)	
Nx	468 (1.40%)	243 (0.80%)	225 (7.68%)	
Median household income^b^				0.597
Low	16,848 (50.37%)	15,358 (50.32%)	1490 (50.84%)	
High	16,601 (49.63%)	15,160 (49.68%)	1441 (49.16%)	
Marital status				0.003
Married	20,482 (61.23%)	18,669 (61.17%)	1813 (61.86%)	
Previous married	5877 (17.57%)	5344 (17.51%)	533 (18.18%)	
Never married	5293 (15.82%)	4822 (15.80%)	471 (16.07%)	
Unknown	1797 (5.37%)	1683 (5.51%)	114 (3.89%)	
Insurance status				<0.001
Insured	31,971 (95.58%)	29,173 (95.59%)	2798 (95.46%)	
Uninsured	1008 (3.01%)	895 (2.93%)	113 (3.86%)	
Unknown	470 (1.41%)	450 (1.47%)	20 (0.68%)	
Lymph node removal				<0.001
No	29,633 (88.59%)	27,324 (89.53%)	2309 (78.78%)	
Yes	3816 (11.41%)	3194 (10.47%)	622 (21.22%)	
Cancer‐directed Surgery				<0.001
No^c^	1991 (5.95%)	722 (2.37%)	1269 (43.30%)	
Yes	31,458 (94.05%)	29,796 (97.63%)	1662 (56.70%)	
Surgical methods				<0.001
No surgery	1991 (5.95%)	722 (2.37%)	1269 (43.30%)	
Local tumor excision/destruction	1293 (3.87)	1280 (4.21)	13 (0.44)	
Partial nephrectomy	11,267 (33.68)	11,207 (36.84)	60 (2.05)	
Radical nephrectomy	18,471 (55.22)	16,963 (55.77)	1508 (51.45)	
Nephrectomy, NOS	380 (1.14)	305 (0.67)	75 (2.56)	
Surgery, NOS	47 (0.14)	41 (0.13)	6 (0.20)	

Data were n (%), unless otherwise specified.

Abbreviations: DM, distant metastasis; NOS, not otherwise specified; SD, standard deviation; y, years.

^a^ Grade I = Well differentiated; Grade II = Moderately differentiated; Grade III = Poorly differentiated; Grade IV = Undifferentiated.

^b^ Median household income: defined by earnings above the median of the median household income in this sample.

^c^ Including “no surgical procedure,” “needle, or aspiration biopsy,” or “Non‐cancer directed surgery.”

Lung (6.19%) was the most common metastatic site, and brain (1.20%) was the least frequent metastatic organ. Moreover, 3.07% patients had two or more metastatic sites (data were not shown). As exhibited in Table 2, patients with multiple metastatic sites had later tumor stage (T4: 15.69% vs. 9.76%, *p* < 0.001. N1: 29.92% vs. 21.21%, *p* < 0.001) when compared with those with single metastatic site. As for therapies, patents with single metastatic organ had higher proportion of surgery (66.25% vs. 38.99%, *p* < 0.001) and lymph node removal (24.93% vs. 14.33%, *p* < 0.001). However, no significant difference was found in the comparisons of other variables. Finally, patients with single metastatic site were further divided into four groups (brain alone, bone alone, liver alone, and lung alone), and comparisons between groups are shown in Table S1. Patients with lung metastasis received more often surgery (*p* < 0.001) and lymph node removal (*p* < 0.001) when compared with other sites.

**TABLE 2:** Baseline characteristics of patients with DM, stratified by the number of metastatic sites

	1 site	>1 site	*p* value
N	1905	1026	
Age, y, Mean ± SD	62.63 ± 10.80	61.66 ± 10.72	0.0203
<65	1105 (58.01%)	619 (60.33%)	0.222
≥65	800 (41.99%)	407 (39.67%)	
Sex			0.255
Male	1332 (69.92%)	738 (71.93%)	
Female	573 (30.08%)	288 (28.07%)	
Race			0.142
White	1642 (86.19%)	857 (83.53%)	
Black	107 (5.62%)	79 (7.70%)	
Other	150 (7.87%)	86 (8.38%)	
Unknown	6 (0.31%)	4 (0.39%)	
Laterality			0.755
Left	964 (50.60%)	513 (50.00%)	
Right	941 (49.40%)	513 (50.00%)	
Grade^a^			<0.001
Grade I	40 (2.10%)	22 (2.14%)	
Grade II	336 (17.64%)	113 (11.01%)	
Grade III	583 (30.60%)	234 (22.81%)	
Grade IV	378 (19.84%)	166 (16.18%)	
Unknown	568 (29.82%)	491 (47.86%)	
T stage			<0.001
T1	350 (18.37%)	121 (11.79%)	
T2	332 (17.43%)	211 (20.57%)	
T3	891 (46.77%)	410 (39.96%)	
T4	186 (9.76%)	161 (15.69%)	
Tx	146 (7.66%)	123 (11.99%)	
N stage			<0.001
N0	1382 (72.55%)	613 (59.75%)	
N1	404 (21.21%)	307 (29.92%)	
Nx	119 (6.25%)	106 (10.33%)	
Median household income^b^			0.413
Low	979 (51.39%)	511 (49.81%)	
High	926 (48.61%)	515 (50.19%)	
Marital status			0.487
Married	1182 (62.05%)	631 (61.50%)	
Previous married	350 (18.37%)	183 (17.84%)	
Never married	294 (15.43%)	177 (17.25%)	
Unknown	79 (4.15%)	35 (3.41%)	
Insurance status			0.359
Insured	1822 (95.64%)	976 (95.13%)	
Uninsured	68 (3.57%)	45 (4.39%)	
Unknown	15 (0.79%)	5 (0.49%)	
Lymph node removal			<0.001
No	1430 (75.07%)	879 (85.67%)	
Yes	475 (24.93%)	147 (14.33%)	
Cancer‐directed Surgery			<0.001
No^c^	643 (33.75%)	626 (61.01%)	
Yes	1262 (66.25%)	400 (38.99%)	

Data were n (%), unless otherwise specified.

Abbreviations: DM, distant metastasis; SD, standard deviation; y, years.

^a^ Grade I = Well differentiated; Grade II = Moderately differentiated; Grade III = Poorly differentiated; Grade IV = Undifferentiated.

^b^ Median household income: defined by earnings above the median of the median household income in this sample.

^c^ Including “no surgical procedure,” “needle, or aspiration biopsy,” or “Non‐cancer directed surgery.”

### 3.2 Incidence of distant metastasis

**FIGURE 2:**
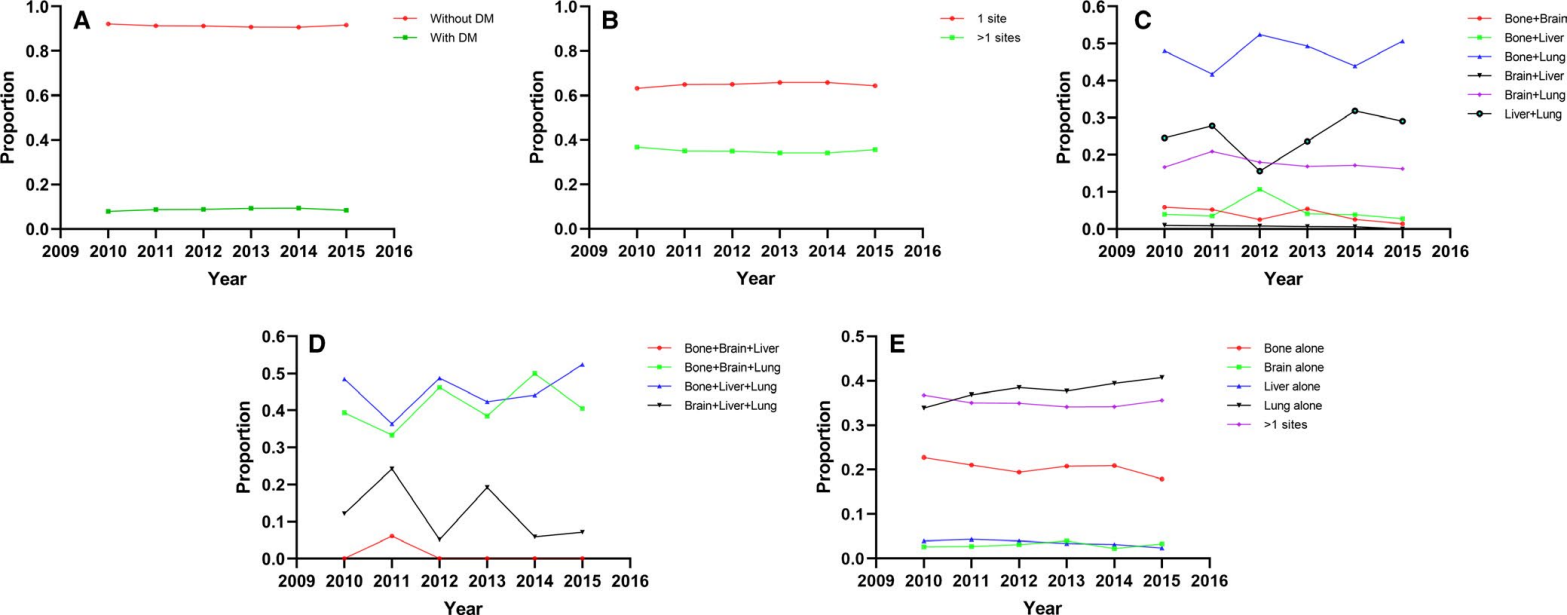
Proportion trends of metastatic patients in ccRCC patients (A). Proportion trends of different metastatic patterns: the number of metastatic sites (B), patients with two sites (C), patients with three sites, (D) one site or more than one site (E)

To explore the proportion trends of metastatic patients in the total ccRCC patients, the proportion trends of patients with single or multiple metastatic sites in the metastatic patients, we developed the related time line charts. Proportion trends of metastatic patients in ccRCC patients is shown in Figure 2A. Moreover, proportion trends of different metastatic patterns are shown in Figure 2B‐E: the number of metastatic sites (Figure 2B), patients with two sites (Figure 2C), patients with three sites (Figure 2D), and one site vs. more than one site (Figure 2E). All the line charts tended to be stable from 2010 to 2015, except for those with multiple metastatic sites, especially for three sites. We attributed the large fluctuation of some specific curves to the small population of these groups, and a slight change in the number of patients would lead to a larger change in the rate.

### 3.3 Proportional mortality ratio

**FIGURE 3:**
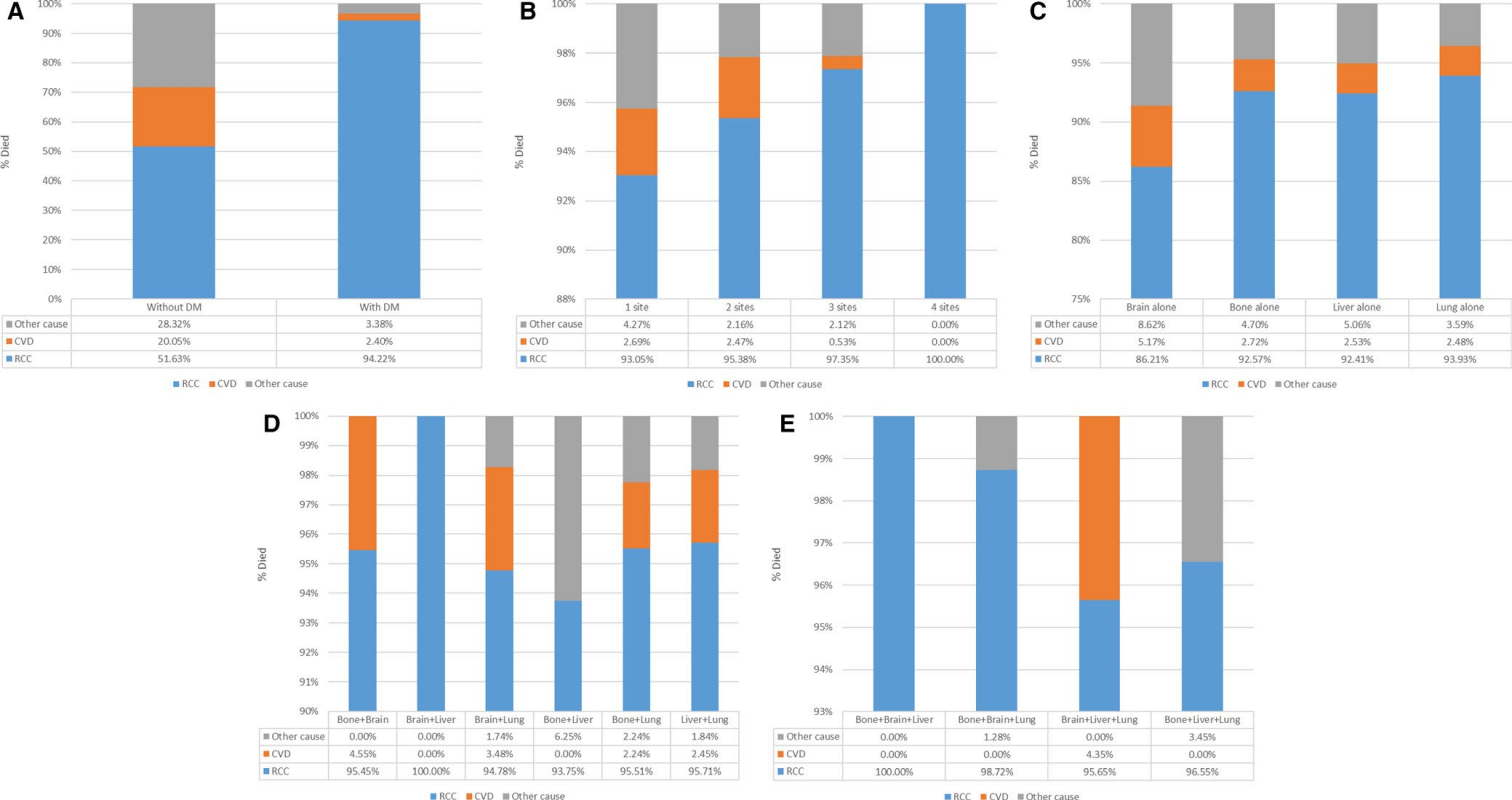
PMRs of ccRCC patients (A), and PMRs of patients with DM: the number of metastatic sites (B), patients with one site (C), patients with two sites (D), patients with three sites (E)

In this study, a total of 5745 patients died up to 31 December 2017, including 3616 nonmetastatic patients and 2129 metastatic patients. PMRs were as follows: RCC 67.4% (3873/5745), CVD 13.5% (776/5745), and other causes 19.1% (1096/5745). Figure 3 showed the outcomes of subgroup analyses. Conclusions could be drawn that in patients with DM, the proportion of death from RCC increased significantly (51.63%–94.22%), while the proportion of death from CVD (20.05%–2.40%) and other causes (28.32%–3.38%) decreased significantly when compared with patients without DM (Figure 3A). That was, once the patient had DM, it was extremely likely to die from the disease itself. With the increase of metastatic sites, this trend became more obvious (Figure 3B). The distribution of PMRs in patients with the same number of metastases did not change significantly (Figure 3C–E). Furthermore, in patients with three or four metastases, the proportion of death from RCC can be as high as 100% (Figure 3E).

### 3.4 Survival outcomes

**FIGURE 4:**
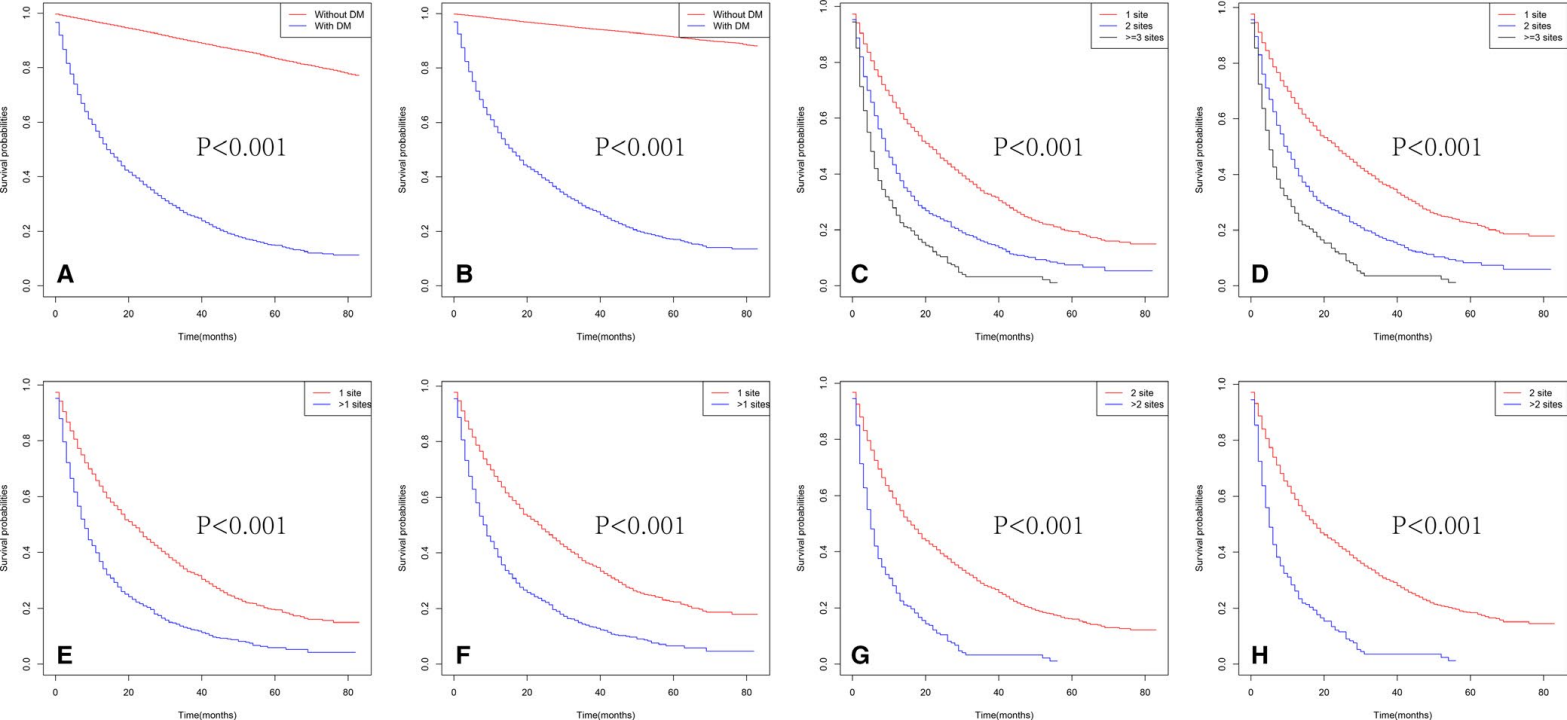
Kaplan–Meier curves of OS in patients according to metastatic status: with or without DM (A), the number of metastatic sites (C), 1 site versus >1 sites (E), 2 sites versus >2 sites (G). Kaplan–Meier curves of CSS in patients according to metastatic status: with or without DM (B), the number of metastatic sites (D), 1 site versus >1 sites (F), 2 sites versus >2 sites (H)

**FIGURE 5:**
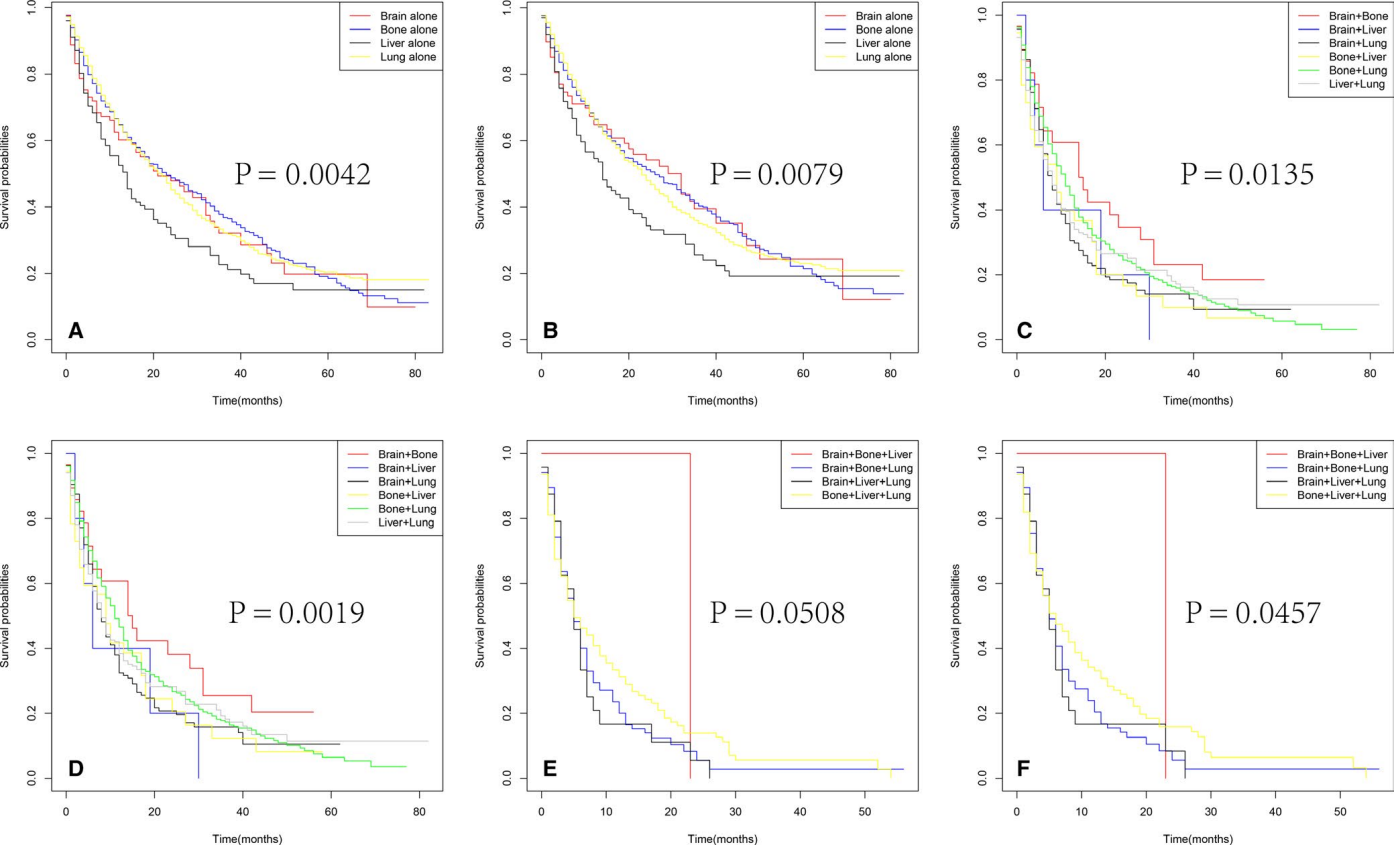
Kaplan–Meier curves of OS in patients according to metastatic status: with single site (A), with two sites (C), with three sites (E). Kaplan–Meier curves of CSS in patients according to metastatic status: with single site (B), with two sites (D), with three sites (F)

As shown in Figure 4A,B, in terms of OS, patients without DM had better OS and CSS than those with DM. Besides, as the number of metastases increased in patients with DM, the long‐term OS, and CSS probabilities decreased significantly (Figure 4C, D). In addition, patients with multiple metastatic sites had worse OS and CSS than those with single metastatic site (Figure 4E, F), and the same in the comparisons between two metastatic sites and more (Figure 4G, H). Among all metastatic sites, those with liver metastasis had the worst OS and CSS (Figure 5A, B). Moreover, survival curves of patients with two or more metastatic sites are shown in Figure 5C, D and Figure 5E, F, respectively. And no significant differences were identified in OS (Figure 5E, *p* = 0.0508) and CSS (Figure 5F, *p* = 0.0457) for various metastatic patterns among patients with three or more metastatic sites.

As shown in Table 3, ccRCC patients with higher tumor grade (Grade III: odds ratio (OR) = 2.538, Grade IV: OR = 4.694, all *p* < 0.001), T stage (T2: OR = 7.177, T3: OR = 7.964, T4: OR = 34.118, all *p* < 0.001), and N stage (N1: OR = 5.873, *p* < 0.001) had a higher risk of DM. In patients with DM, age at diagnosis (hazard ratio (HR) = 1.206, *p* = 0.004), grade (Grade IV: HR = 2.256, *p* < 0.001), T stage (T4: HR = 1.461, *p* = 0.003), N stage (HR = 1.576, *p* < 0.001), the administration of surgery (HR = 0.380, *p* < 0.001), the number of metastatic sties (2 sites: HR = 1.418, *p* < 0.001. ≥3 sites: HR = 2.552, *p* < 0.001), and marital status (Previous married: HR = 1.240, *p* = 0.006) were important factors affecting the OS (Table 4), and grade (Grade III: HR = 1.693, *p* = 0.019. Grade IV: HR = 2.436, *p* < 0.001), T stage (T3: HR = 1.248, *p* = 0.044. T4: HR = 1.544, *p* = 0.001), N stage (HR = 1.558, *p* < 0.001), the administration of surgery (HR = 0.370, *p* < 0.001), the number of metastatic sties (2 sites: HR = 1.479, *p* < 0.001. ≥3 sites: HR = 2.695, *p* < 0.001), marital status (previous married: HR = 1.249, *p* = 0.006), and household income (HR = 0.842, *p* = 0.001) were significantly related to CSS (Table 5).

**TABLE 3:** Univariate and multivariate logistic regression analyses of risk factors for patients with DM

Variable	Univariate			Multivariate		
	OR	95% CI	*p* value	OR	95% CI	*p* value
Age			0.850			
<65	Reference					
≥65	0.990	0.893–1.098	0.850			
Race			**0.027**			0.151
White	Reference			Reference		
Black	0.777	0.626–0.965	0.023	0.861	0.675–1.100	0.231
Other	1.222	0.936–1.346	0.214	1.161	0.947–1.424	0.150
Sex			**<0.001**			0.234
Male	Reference			Reference		
Female	0.675	0.605–0.752	<0.001	0.928	0.821–1.049	0.234
Grade^a^			**<0.001**			**<0.001**
Grade I	Reference			Reference		
Grade II	1.862	1.360–2.550	<0.001	1.315	0.951–1.820	0.098
Grade III	6.908	5.079–9.394	<0.001	2.538	1.838–3.504	<0.001
Grade IV	24.848	18.139–34.037	<0.001	4.694	3.355–6.566	<0.001
Laterality			0.087			
Left	Reference					
Right	0.917	0.830–1.013	0.087			
T stage			**<0.001**			**<0.001**
T1	Reference			Reference		
T2	9.991	8.349–11.955	<0.001	7.177	5.965–8.636	<0.001
T3	15.203	13.071–17.683	<0.001	7.964	6.763–9.379	<0.001
T4	93.577	73.210–119.612	<0.001	34.118	26.023–44.733	<0.001
N stage			**<0.001**			**<0.001**
N0	Reference			Reference		
N1	20.801	17.852–24.237	<0.001	5.873	4.959–6.957	<0.001
Insurance status			0.280			
Insured	Reference					
Uninsured	1.160	0.886–1.519	0.280			
Marital status			0.347			
Married	Reference					
Previous married	0.959	0.840–1.094	0.533			
Never married	0.904	0.785–1.040	0.158			
Household income^b^			0.664			
Low	Reference					
High	0.978	0.885–1.081	0.664			

The bold value means that the corresponding p of the variable is less than 0.05, with statistical significance.

Abbreviations: CI, confidence interval; DM, distant metastasis; OR, odds ratio.

^a^ Grade I = Well differentiated; Grade II = Moderately differentiated; Grade III = Poorly differentiated; Grade IV = Undifferentiated

^b^ Median household income: defined by earnings above the median of the median household income in this sample.

**TABLE 4:** Univariate and Multivariate Cox regression analyses of prognostic factors for OS in patients with DM

Variable	Univariate			Multivariate		
	HR	95% CI	*p* value	HR	95% CI	*p* value
Age			**0.027**			**0.004**
<65	Reference			Reference		
≥65	1.147	1.016–1.296	0.027	1.206	1.063–1.368	0.004
Race			0.717			
White	Reference					
Black	1.012	0.781–1.312	0.926			
Other	0.914	0.734–1.138	0.422			
Sex			0.233			
Male	Reference					
Female	1.082	0.951–1.231	0.233			
Grade^a^			**<0.001**			**<0.001**
Grade I	Reference			Reference		
Grade II	1.068	0.702–1.625	0.758	1.259	0.823–1.924	0.288
Grade III	1.192	0.791–1.797	0.401	1.610	1.055–2.455	0.207
Grade IV	1.651	1.092–2.495	0.017	2.256	1.466–3.472	<0.001
Laterality			0.661			
Left	Reference					
Right	0.974	0.865–1.096	0.661			
T stage			**<0.001**			**0.003**
T1	Reference			Reference		
T2	1.273	1.014–1.598	0.037	0.993	0.789–1.251	0.955
T3	1.396	1.150–1.695	0.001	1.176	0.960–1.439	0.117
T4	2.256	1.780–2.859	<0.001	1.461	1.138–1.874	0.003
N stage			**<0.001**			**<0.001**
N0	Reference			Reference		
N1	2.015	1.767–2.297	<0.001	1.576	1.374–1.808	<0.001
Surgery			**<0.001**			**<0.001**
No^b^	Reference			Reference		
Yes	0.373	0.324–0.430	<0.001	0.380	0.322–0.447	<0.001
Lymph node removal			0.744			
No	Reference					
Yes	1.021	0.901–1.158	0.744			
Number of metastatic sites			**<0.001**			**<0.001**
1 site	Reference			Reference		
2 sites	1.810	1.576–2.079	<0.001	1.418	1.284–1.709	<0.001
≥3 sites	3.474	2.760–4.372	<0.001	2.552	2.014–3.233	<0.001
Insurance status			0.737			
Insured	Reference					
Uninsured	1.056	0.770–1.447	0.737			
Marital status			**0.001**			**0.020**
Married	Reference			Reference		
Previous married	1.332	1.147–1.547	<0.001	1.240	1.064–1.445	0.006
Never married	1.086	0.917–1.286	0.341	1.005	0.846–1.193	0.958
Median household income^c^			0.113			
Low	Reference					
High	0.908	0.807–1.023	0.113			

The bold value means that the corresponding p of the variable is less than 0.05, with statistical significance.

Abbreviations: CI, confidence interval; DM, distant metastasis; HR, hazard ratio; OS, overall survival.

^a^ Grade I = Well differentiated; Grade II = Moderately differentiated; Grade III = Poorly differentiated; Grade IV = Undifferentiated.

^b^ Including “no surgical procedure,” “needle, or aspiration biopsy,” or “Non‐cancer directed surgery.”

^c^ Median household income: defined by earnings above the median of the median household income in this sample.

**TABLE 5:** Univariate and Multivariate Cox regression analyses of prognostic factors for CSS in patients with DM

Variable	Univariate			Multivariate		
	OR	95% CI	*p* value	OR	95% CI	*p* value
Age			0.137			
<65	Reference					
≥65	1.101	0.970–1.250	0.137			
Race			0.567			
White	Reference					
Black	0.958	0.728–1.261	0.758			
Other	0.885	0.702–1.115	0.299			
Sex			0.323			
Male	Reference					
Female	1.070	0.936–1.224	0.323			
Grade^a^			**<0.001**			**<0.001**
Grade I	Reference			Reference		
Grade II	1.028	0.662–1.594	0.903	1.275	0.818–1.987	0.283
Grade III	1.195	0.778–1.834	0.416	1.693	1.089–2.632	0.019
Grade IV	1.753	1.140–2.697	0.011	2.436	1.553–3.820	<0.001
Laterality			0.602			
Left	Reference					
Right	0.968	0.856–1.094	0.602			
T stage			**<0.001**			**0.002**
T1	Reference			Reference		
T2	1.349	1.060–1.717	0.015	1.042	0.816–1.330	0.742
T3	1.501	1.220–1.847	<0.001	1.248	1.006–1.548	0.044
T4	2.463	1.920–3.161	<0.001	1.544	1.188–2.005	0.001
N stage			**<0.001**			**<0.001**
N0	Reference			Reference		
N1	2.046	1.786–2.343	<0.001	1.558	1.352–1.795	<0.001
Surgery			**<0.001**			**<0.001**
No^b^	Reference			Reference		
Yes	0.379	0.327–0.439	<0.001	0.370	0.312–0.439	<0.001
Lymph node removal			0.406			
No	Reference					
Yes	1.056	0.928–1.203	0.406			
Number of metastatic sites			**<0.001**			**<0.001**
1 site	Reference			Reference		
2 sites	1.834	1.597–2.126	<0.001	1.479	1.275–1.714	<0.001
≥3 sites	3.645	2.886–4.604	<0.001	2.695	2.119–3.429	<0.001
Insurance status			0.839			
Insured	Reference					
Uninsured	1.035	0.742–1.444	0.839			
Marital status			**0.003**			**0.012**
Married	Reference			Reference		
Previous married	1.308	1.120–1.528	0.001	1.249	1.066–1.463	0.006
Never married	1.067	0.893–1.274	0.475	0.958	0.802–1.146	0.641
Median household income^c^			**0.030**			**0.007**
Low	Reference			Reference		
High	0.872	0.771–0.987	0.030	0.842	0.743–0.954	0.007

The bold value means that the corresponding *p* of the variable is less than 0.05, with statistical significance.

Abbreviations: CI, confidence interval; CSS, cancer‐specific survival; DM, distant metastasis; HR, hazard ratio.

^a^ Grade I = Well differentiated; Grade II = Moderately differentiated; Grade III = Poorly differentiated; Grade IV = Undifferentiated.

^b^ Including “no surgical procedure,” “needle, or aspiration biopsy,” or “Non‐cancer directed surgery”.

^c^ Median household income: defined by earnings above the median of the median household income in this sample.

Similarly, in patients with single metastatic site, multivariate Cox analysis showed that: age at diagnosis (HR = 1.212, *p* = 0.014), grade (Grade III: HR = 2.131, *p* = 0.015. Grade IV: HR = 3.039, *p* < 0.001), T stage (T3: HR = 1.313, *p* = 0.029. T4: HR = 1.814, *p* < 0.001), N stage (HR = 1.735, *p* < 0.001), the administration of surgery (HR = 0.367, *p* < 0.001), and marital status (HR = 1.324, *p* = 0.003) were significant factors associated with OS (Table S2). Age at diagnosis (HR = 1.185, *p* = 0.037), Grade (Grade III: HR = 2.157, *p* = 0.019. Grade IV: HR = 3.228, *p* < 0.001), T stage (T3: HR = 1.381, *p* = 0.015. T4: HR = 1.915, *p* < 0.001), N stage (HR = 1.749, *p* < 0.001), the administration of surgery (HR = 0.369, *p* < 0.001), marital status (Previous married: HR = 1.303, *p* = 0.007), and household income (HR = 0.853, *p* = 0.045) were significantly related to CSS (Table S3).

## 4 DISCUSSION

In our study, we found that 8.76% of ccRCC patients had DM at the time of diagnosis, and 3.07% of patients suffered multiple metastases. Lung (6.19%, 2070/33,44) was the most common organ of metastasis, followed by bone (3.74%, 1251/33,449). In addition, we found that the proportion of all metastatic patterns showed stable trends with no obvious changes in recent years. However, the metastatic rate in this study was significantly lower than 18%–30% reported in previous studies.^6^, ^7^ We attributed this to the fact that we only included patients with distant organs metastases, while regional (did not meet the inclusion criteria) and distant (due to the limitation of the database itself) lymph node metastases (LNM) were excluded. Moreover, only ccRCC was included in this study, while some other pathological types had high potential for malignant metastasis, such as Bellini duct carcinoma and medullary carcinoma. Therefore, it was believed that this data only represented ccRCC, which was more convincing. Last but not least, with the understanding of disease and the rapid improvement of diagnostic technology, increasing early stage RCC or small renal mass were found in clinical work.

By far, there were only few studies focused on the combined metastatic patterns of RCC. However, 35.01% (1026/2931) of the metastatic patients suffered multiple metastases in our study. In these patients, lung plus bone (36.84%) was the most common co‐metastases type, followed by lung plus liver (19.8%) and lung plus brain (13.5%). Furthermore, patients with multiple metastases had worse survival outcomes than those with single metastatic site, and the prognosis was getting worse with the increase of the number of metastatic sites (Tables 4 and 5). Hence, it was of great importance to examine the possibility of combined metastases, by which we can fully grasp the progress of the disease and make individualized treatment plans. However, comparisons of prognosis in patients with three of more metastatic sites were not different significantly, which may due to the fact that the prognosis of all these patients was very poor.

Nowadays, CVDs are the leading causes of mortality worldwide.^19^ It was reported that there were 17.7 million deaths because of CVDs and 8.8 million deaths due to cancer worldwide in 2015.^20^ Sturgeon et al.^20^ investigated CVD mortality risk in cancer patients, and demonstrated that cancer patients with higher risk of dying from CVDs when compared with the general population. Ward et al.^21^ proposed that CVD was the leading cause of death among endometrial cancer patients. Mehta^22^ discussed that CVD resulted in heavier burden than breast cancer itself in older women. Previous studies have reported that smoking,^23^, ^24^ obesity,^25^, ^26^ and hypertension^27^ were risk factors of RCC, and thus, we discussed CVD mortality in the analysis of PMRs. In our study, we found that the PMR was 20.05% in patients without DM died due to CVDs, while the PMR was only 2.4% in patients with DM. Moreover, with the increase of the number of metastatic sites, the PMR of CVD decreased accordingly and tended to be zero. Consequently, although patients with RCC should pay special attention to CVD, they should be more committed to the treatment of the disease itself in case of metastasis.

Survival analysis showed that the prognosis of patients with DM was significantly worse. In addition, as mentioned above, the prognosis was worse as the increase of the number of metastatic sites. Therefore, it was extremely urgent to identify the risk factors of DM and the prognostic factors for metastatic patients. Multivariate logistic regression analysis revealed that higher tumor grade, T stage, and N stage were important risk factors for DM. A meta‐analysis carried out by Thompson et al.^28^ showed that poor differentiation was a risk factor for metastasis of cutaneous squamous cell carcinoma. Zhang et al.^29^ discovered that higher T stage, higher N stage, and poor tumor differentiation grade were positively related to bone metastases in initial bladder cancer. Similar conclusion was drawn in the study conducted by Moon.^30^ In patients with DM, multivariate Cox analysis showed that tumor grade, age at diagnosis, T stage, N stage, marital status, and the administration of surgery was a prognostic factor for OS. Many previous studies have found that age at diagnosis, T stage, N stage, and tumor differentiation played important roles in cancer survival outcomes.^31^, ^32^, ^33^ Moreover, CN has been recognized to have survival benefits in mRCC patients. As for CSS, higher median household income was associated with better prognosis. Daniel Lin et al.^34^ found that median household income may be an independent predictor for CSS in patients with squamous cell carcinoma of the anus. However, Torbrand et al.^35^ considered that socioeconomic status influenced the stage and risk but not survival outcomes in patients with penile cancer.

However, there were some limitations that should not be ignored in our study. First of all, we did not included patients with distant LNM because the related data were available in the database after 2016. Second, the sequence of metastases could not be known for patients with multiple metastases, which may be an important obstacle to carry out further exploration. Furthermore, there were few patients in some specific distant patterns, and the epidemiological tendency and survival outcomes may be influenced. Additionally, several important factors are lacking in the SEER database, including LDH, hemoglobin, neutrophil count platelet count, MSKCC or IMDC risk classification, and so on. Finally, it was a retrospective and database‐based research, further studies with large sample size and detailed related information are needed in the future.

## 5 CONCLUSION

About 8.76% of ccRCC patients suffered DM at their initial diagnosis, among them 35.01% of the patients with multiple metastases. Patients with DM had poor survival outcomes than those without DM, and decreased survival was identified in patients with increased number of metastatic sites. Furthermore, predictive and prognostic factors of DM were then investigated to provide potential values in clinical guidance.

## AUTHORS’ CONTRIBUTIONS

FQ and ZW conceived and designed the study. JX, WC, and WX collected the data. JX, WC, and ZX analyzed the data. JX, WC, and XL provided the resources for the study. ZW supervised the study. JX, WC, and FQ wrote the manuscript. All authors read and approved the final manuscript prior to submission.

## ETHICS APPROVAL AND CONSENT PARTICIPATE

As the data used were from SEER data set (public). Ethics approval and consent to participate could be checked in SEER.

## Data Availability

All data included in this study are available on reasonable request from the corresponding author.
